# Association of clinical prediction scores with hospital mortality in an adult medical and surgical intensive care unit in Kenya

**DOI:** 10.3389/fmed.2023.1127672

**Published:** 2023-04-05

**Authors:** B. Jason Brotherton, Mugdha Joshi, George Otieno, Sarah Wandia, Hannah Gitura, Ariel Mueller, Tony Nguyen, Steve Letchford, Elisabeth D. Riviello, Evelyn Karanja, Kristina E. Rudd

**Affiliations:** ^1^Department of Internal Medicine, AIC Kijabe Hospital, Kijabe, Kenya; ^2^The Clinical Research, Investigation, and Systems Modeling of Acute Illness (CRISMA) Center, Department of Critical Care Medicine, University of Pittsburgh, Pittsburgh, PA, United States; ^3^Department of Medicine, Stanford University, Palo Alto, CA, United States; ^4^Department of Emergency and Critical Care Medicine, AIC Kijabe Hospital, Kijabe, Kenya; ^5^Department of Anesthesia, Critical Care and Pain Medicine, Massachusetts General Hospital, Boston, MA, United States; ^6^Division of Pulmonary, Critical Care and Sleep Medicine, Beth Israel Deaconess Medical Center, Harvard Medical School, Boston, MA, United States

**Keywords:** global health, critical care, mortality prediction, resource variable, severity of illness, Kenya

## Abstract

**Importance:**

Mortality prediction among critically ill patients in resource limited settings is difficult. Identifying the best mortality prediction tool is important for counseling patients and families, benchmarking quality improvement efforts, and defining severity of illness for clinical research studies.

**Objective:**

Compare predictive capacity of the Modified Early Warning Score (MEWS), Universal Vital Assessment (UVA), Tropical Intensive Care Score (TropICS), Rwanda Mortality Probability Model (R-MPM), and quick Sequential Organ Failure Assessment (qSOFA) for hospital mortality among adults admitted to a medical-surgical intensive care unit (ICU) in rural Kenya. We performed a pre-planned subgroup analysis among ICU patients with suspected infection.

**Design, setting, and participants:**

Prospective single-center cohort study at a tertiary care, academic hospital in Kenya. All adults 18 years and older admitted to the ICU January 2018–June 2019 were included.

**Main outcomes and measures:**

The primary outcome was association of clinical prediction tool score with hospital mortality, as defined by area under the receiver operating characteristic curve (AUROC). Demographic, physiologic, laboratory, therapeutic, and mortality data were collected. 338 patients were included, none were excluded. Median age was 42 years (IQR 33–62) and 61% (*n* = 207) were male. Fifty-nine percent (*n* = 199) required mechanical ventilation and 35% (*n* = 118) received vasopressors upon ICU admission. Overall hospital mortality was 31% (*n* = 104). 323 patients had all component variables recorded for R-MPM, 261 for MEWS, and 253 for UVA. The AUROC was highest for MEWS (0.76), followed by R-MPM (0.75), qSOFA (0.70), and UVA (0.69) (*p* < 0.001). Predictive capacity was similar among patients with suspected infection.

**Conclusion and relevance:**

All tools had acceptable predictive capacity for hospital mortality, with variable observed availability of the component data. R-MPM and MEWS had high rates of variable availability as well as good AUROC, suggesting these tools may prove useful in low resource ICUs.

## Introduction

Critical illness is common in resource limited settings, though precise and comprehensive data on the global burden of critical illness are not available (1, 2). The data we do have suggest that sepsis accounts for 1 in 5 deaths globally, with the highest burden in Africa (3). Similarly, mortality from respiratory failure in resource limited settings has ranged between 36 and 72% (4). Mortality prediction scores are an important tool in caring for critically ill patients, but even more so when resources are constrained (5). A severity of illness tool that can be used in resource constrained settings and accurately predicts mortality may be useful in counseling patients and their families, benchmarking for quality improvement, defining severity of illness for clinical research studies, and possibly in allocating resources to those most likely to benefit.

Accurate mortality risk prognostication for critically ill patients in resource constrained settings is challenging. Many illness severity scores developed and validated in intensive care units (ICUs) in high income countries (HICs) have proven to be less useful in some resource-limited settings within low and middle-income countries (LMICs) due to their high volume of physiologic variables and laboratory requirements, resulting in large amounts of missing data (6). This, along with differences in patient population demographics and disease processes, has limited their generalizability and practical relevance to resource-constrained settings (7).

Poor applicability of severity of illness scores developed in HICs to other contexts has led to the modification of more complex scores, and the creation of new scores, for use in resource constrained contexts. Examples are the Modified Early Warning Score (MEWS), Universal Vitals Assessment (UVA), quick Sequential Organ Failure Assessment (qSOFA), Rwanda Mortality Prediction Model (R-MPM), and the Tropical Intensive Care Score (TropICS) (8–12). MEWS, UVA, and qSOFA have previously been compared to one another in their ability to predict mortality in a hospital wide population and among ICU cohorts in low-resource settings (13, 14). However, they have not been compared simultaneously to R-MPM or TropICS. Therefore, our primary objective in this study was to compare the predictive capacity of these tools for hospital mortality in a rural Kenyan ICU. We also performed a pre-specified subgroup analysis comparing the performance of these scores among ICU patients with suspected infection, given that qSOFA was originally designed to be used among populations with suspected infection.

## Materials and methods

This study was approved with a waiver of consent by the AIC Kijabe Hospital (KH) Institutional Review Board.

### Study design, setting, and population

This was a prospective, observational study carried out in KH in Kijabe, Kenya between 1 January 2018, and 30 June 2019. All patients aged 18 years and older admitted to the KH adult ICU during the study period were consecutively enrolled. There were no exclusion criteria aside from age < 18 years.

Admissions to the ICU during the study period came from casualty (i.e., Emergency Department), inpatient acute care medical and surgical wards, and the operating theater, or were transferred from other medical facilities. Individuals participating in the daily care of ICU patients (e.g., consultant physicians, medical officers, clinical officers, and residents) participated in data entry and were solely responsible for all medical decisions. No additional clinical assessments or laboratory tests were performed by the study team or for the purposes of the study.

Kijabe Hospital is a 360-bed academic, tertiary care referral hospital located in rural Kenya. Services available at KH during the study period included internal medicine, general surgery, orthopedic surgery, obstetrics and gynecology, general pediatrics, and pediatric surgery. At the time of this study, as is common in many resource constrained settings, there was no formally trained critical care physician on site (15). However, KH is a training site for a nationally recognized diploma program for clinical officers in emergency and critical care medicine (16, 17). KH has 5 adult ICU beds and 10 adult high dependency unit (HDU) beds. The ICU has 5 functioning mechanical ventilators, continuous cardiopulmonary monitoring, and continuous infusion pumps for the administration of intravenous fluids, vasopressors, and insulin. Vasopressors available include norepinephrine, dopamine, and epinephrine. Non-invasive ventilation was not available at KH during the study period.

### Data collection and exposure variables

Exposure variables were the MEWS, UVA, TropICS, R-MPM, and qSOFA scores at the time of admission to the ICU. Scores for each were determined based on the definitions used in the referenced studies (Supplementary Table 2). The first vital signs upon admission to the ICU were used. A locally created mobile application, Banda Health, was used to collect admission data in real time.^1^ Demographic data and clinical components of each score/model were collected upon ICU admission. In addition, discharge diagnoses and vital status at discharge were collected. If data were found to be missing when downloaded from the mobile application, the paper health records were retrieved to search for the missing variables. If after this attempt data were still missing, the value was left blank in the data set. Missing physiologic variables were then imputed as normal. We chose to assign a normal value for missing data to simulate what would be done in a real-life clinical setting in the instance of missing values. Percentages of missing data per variable are listed in Supplementary Table 1.

### Scores

See Supplementary Table 2 for the variables included in each score, method of calculation for each score, and definition of a “positive” score in those used as binary.

The MEWS score was originally developed to identify medical inpatients that might deteriorate to the point of requiring ICU care (18). The predictive accuracy of the MEWS for hospital mortality has previously been evaluated among hospitalized adults in Uganda and Tanzania (8, 19). MEWS scores range from 0 to 14. Neurologic assessment for MEWS included assessment with AVPU (alert, verbal, pain, unresponsive). To be consistent with the scoring of variables in other tools, we converted AVPU to GCS (20). We defined a MEWS score ≥ 5 as “positive” for mortality prediction, as was previously used in other validation studies in East Africa (8).

The UVA score was developed based upon a retrospective, secondary analysis of data from 12 hospitals in Sub-Saharan Africa (9). Most patients included in the original analysis were ward patients, as ICUs were not common in the centers included in the analysis. UVA is scored 0 to 13 with further stratifications of low (0–1), medium (2–4), or high risk (>4) (9). We similarly defined a “positive” high risk score threshold of >4 in our analyses.

Tropical Intensive Care Score was created using a prospective cohort of patients admitted to ICUs in Sri Lanka, Bangladesh, Nepal, and India (12). In the original manuscript, three different scores were created with increasing numbers of variables involved. Model 2, or TropICS, was found to have the best discrimination and calibration for their cohort. The authors created a nomogram to assign an overall illness score, and another to aid in predicting ICU mortality. We used the same nomogram to assign scores for the six individual variables involved (Supplementary Figure 1). To calculate a score, we chose the median value for each variable increment and then drew a line through the corresponding score line. The individual variable component scores were used to tally total scores, and an associated mortality risk from the corresponding nomogram was assigned based upon this total score.

The R-MPM was created from patient data prospectively collected from two Rwandan ICUs (11). In this study, univariate and multivariate regressions were performed to identify five variables considered to be most predictive of ICU mortality. Using this regression model, study authors found an area under the receiver operator curve of 0.81. Despite using categorical variables, the R-MPM model does not result in a summated score. The regression model must be re-run to elicit a predicted mortality risk for each patient. We used the same variables to generate an AUROC for comparison to the other scores. The R-MPM has never before been validated outside of the original study.

The qSOFA score was introduced with the release of the Sepsis-3 definitions (21). Using three physiologic variables, each scored 0 or 1, this was considered easy to use by clinicians at the bedside to identify, from among patients with suspected infection, those at highest risk for death (21, 22). A score cut-off of ≥2 was initially proposed for mortality prediction. This score has since been evaluated in multiple resource-variable settings, and its sensitivity has been shown to be improved with a threshold score of ≥1 (10, 23). We therefore used a cut-off of ≥1.

### Outcome

The primary outcome of interest was area under the receiver operator curve (AUROC) for all-cause hospital mortality. We performed a pre-planned subgroup analysis among only those patients with suspected infection, as defined by receipt of antibiotics within 24 h of ICU admission, with the hypothesis that qSOFA may perform better among that group of patients given its original development among patients with suspected infection.

### Statistical analysis

Statistical analyses were performed using STATA 15. We calculated proportions for categorical values, and medians with interquartile ranges (IQRs) for continuous variables. To determine the association of categorical variables with mortality, X^2^ or Fischer’s exact test were used. For continuous variables, Wilcoxon rank sum tests were used to determine association with mortality. Because we compared three scores and a logistic regression model, direct comparison using *p*-values was not possible. For consistency we used the same cutoffs for the scores as those in the cited studies. To determine which score or model was most strongly associated with mortality overall we used the area under the receiver operating curve (AUROC).

## Results

During the study period 338 patients were admitted to the ICU, and all were included in this study (Table 1). The median age was 42 years (IQR 33–62), and 61% (*n* = 207) were male. Overall hospital mortality was 31% (*n* = 104). Shock was present on admission in 118 patients (35%), and 199 (59%) were mechanically ventilated. Just over 35% were medical patients (*n* = 120), and the remaining patients were admitted to one of several surgical services. Almost half (48%, *n* = 163) received antibiotics within 24 h of ICU admission and were thus considered to have suspected infection. Over one third (36%, *n* = 122) were admitted to the ICU following emergent surgery. Receipt of mechanical ventilation (89% of those who died were mechanically ventilated versus 46% of those who survived), receipt of vasopressors (72% of those who died received them vs. 18% of those who survived), and presence of suspected infection (65% of those who died had infection vs. 41% of those who survived) were all associated with mortality (*p* < 0.001 for all).

**TABLE 1:** Patient characteristics.

	All patients *N* = 338	Survived *N* = 234	Died *N* = 104	*p*-value
Age (years), median (IQR)	42 (33–62)	45 (34–62)	42 (30–61)	0.4
Male gender, *n* (%)	207 (61)	147 (63)	60 (58)	0.4
**Initial vital signs upon ICU admission, median (IQR)**				
Temperature (°C)	37 (36.5–37)	37 (36.5–37)	37 (36.4–37.3)	0.4
Heart rate (beats/min)	100 (82–118)	95 (82–112)	114 (85–125)	<0.001
Respiratory rate (breaths/min)	18 (14–25)	18 (14–22)	22 (15–30)	<0.001
SBP (mmHg)	120 (110–135)	120 (112–136)	120 (94–125)	<0.001
SpO2 (%)	96 (92–99)	96 (92–99)	96 (91–99)	0.4
GCS	11 (3–15)	13 (7–15)	4 (3–13)	<0.001
**Initial laboratory results upon ICU admission, median (IQR)**				
Hb (g/dL)	13 (10–13)	13 (11–13)	13 (10–13)	0.5
BUN (mg/dL)	15 (15)	15 (15)	15 (15–29)	0.02
Cr (mg/dL)	1.0 (0.9–1.3)	1.0 (0.9–1.2)	1.0 (0.9–1.6)	0.7
HIV positive, *n* (%)	18 (5.3)	10 (4.3)	8 (7.7)	0.2
**Initial clinical management, n(%)**				
Mechanical ventilation upon ICU admission, *n* (%)	199 (59)	107 (46)	92 (89)	<0.001
Receiving vasopressors upon ICU admission, *n* (%)	118 (35)	43 (18)	75 (72)	<0.001
Received antibiotics within 24 h of ICU admission, *n* (%)	163 (48)	95 (41)	68 (65)	<0.001
Emergency surgery as reason for ICU admission, *n* (%)	122 (36)	86 (37)	36 (35)	0.7

Percentages were calculated with the column total as the denominator. IQR, Interquartile range; SBP, Systolic blood pressure; SpO2, Oxygen saturation; GCS, Glasgow coma scale; Hb, Hemoglobin; BUN, Blood urea nitrogen; Cr, Creatinine; HIV, Human immunodeficiency virus.

Table 2 shows the distribution of the scores and their association with hospital mortality. 331 of 338 patients had available data for all component variables of R-MPM, 261 for MEWS, 253 for UVA, 54 for TropICS, and 262 for qSOFA. Due to the large number of missing variables for TropICS we removed this score from further comparison. The median MEWS score for the overall cohort was 4 (IQR 2–6) while those who died had a median score of 6 (IQR 5–8). One hundred forty-one patients (42%) had a score of ≥5 points, which was associated with increased risk of hospital mortality (OR 6.1, 95% CI 3.7–10.2, *p* < 0.001). Median UVA score was 4 (IQR 2–6) overall, while those that died had a median score of 5 (IQR 4–6). UVA risk category of low (0–1 point), medium (2–4 points), and high (>4 points) were all associated with mortality (*p* < 0.001, 0.02, and <0.001, respectively). High UVA (>4 points) had an OR for death of 4.7 (95% CI, 2.9–7.7). The median qSOFA score was 1 (IQR 1–2) overall and 2 (IQR 1–2) in those who died. A positive qSOFA score (≥1 point) was associated with mortality (OR 5.8, 95% CI, 2.0–16.6, *p* < 0.001).

**TABLE 2:** Distribution of mortality scores and association with hospital mortality.

Predictive score or model	All patients *N* = 338	Survived *N* = 234	Died *N* = 104	*p*-value
**MEWS**				
Median (IQR)	4 (2–6)	3 (2–5)	6 (4–8)	<0.001
≥5 points, *n* (%)	141 (42)	67 (29)	74 (71)	<0.001
**UVA**				
Median (IQR)	4 (2–6)	4 (0–5)	5 (4–6)	<0.001
Low risk (0–1 points), *n* (%)	74 (22)	67 (29)	7 (6.7)	<0.001
Medium risk (2–4 points), *n* (%)	136 (40)	104 (44)	32 (31)	0.02
High risk (>4 points), *n* (%)	128 (38)	63 (27)	65 (63)	<0.001
**qSOFA**				
Median (IQR)	1 (1–2)	1 (1–1)	2 (1–2)	<0.001
0 points, *n* (%)	48 (14)	44 (19)	4 (3.8)	<0.001
≥1 point, *n* (%)	290 (86)	190 (81)	100 (96)	<0.001

Percentages were calculated with the column total as the denominator. R-MPM does not have a score as it is a model that requires regression calculation. IQR, Interquartile range; MEWS, Modified early warning score; UVA, Universal vitals assessment; qSOFA, quick sequential organ failure assessment; R-MPM, Rwanda mortality prediction model.

**FIGURE 1:**
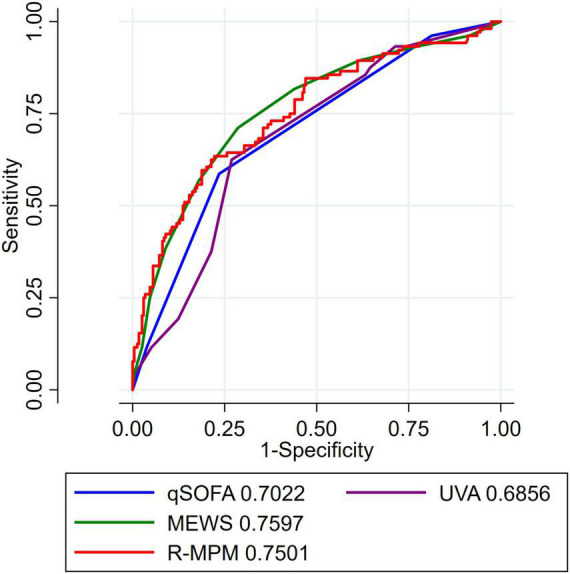
AUROC for all patients. Each tool had a varying number of data missing that is further detailed in Supplementary Table 1. AUROC, area under the receiver operating characteristic curve; MEWS, Modified Early Warning Score; UVA, Universal Vitals Assessment; qSOFA, quick Sequential Organ Failure Assessment; R-MPM, Rwanda Mortality Prediction Model.

Area under the receiver operating characteristic curve was used to compare all four scores with the R-MPM regression model (Figure 1). MEWS had the greatest AUROC with a value of 0.76 (95% CI 0.70–0.81), followed by R-MPM at 0.75 (95% CI 0.69–0.81). The remaining were qSOFA with an AUROC of 0.70 (95% CI 0.65–0.76) and UVA with an AUROC of 0.69 (0.64–0.75).

**FIGURE 2:**
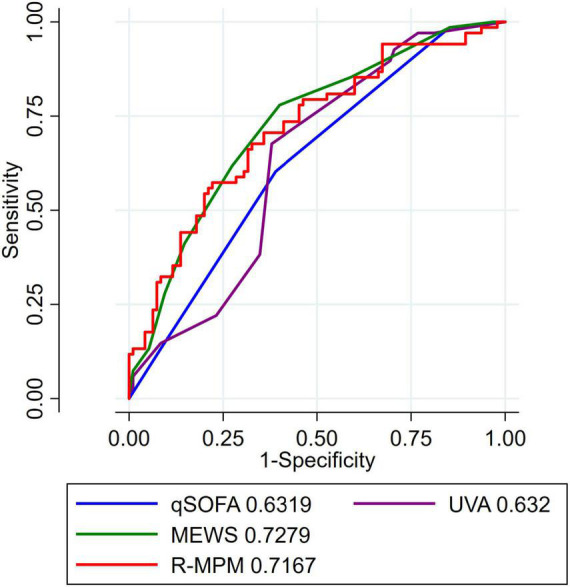
AUROC for patients with suspected infection. AUROC for patients with suspected infection, defined as receipt of IV antibiotics within 24 h of ICU admission. AUROC, area under the receiver operating characteristic curve; MEWS, Modified Early Warning Score; UVA, Universal Vitals Assessment; qSOFA, quick Sequential Organ Failure Assessment; R-MPM, Rwanda Mortality Prediction Model.

In the pre-planned subgroup analysis, we compared the scores and model among only those patients with a suspected infection at the time of ICU admission. There were 163 patients included in this analysis (Table 3). qSOFA score ≥ 1, MEWS ≥ 5, high-risk UVA score, and R-MPM were all associated with increased risk of death in those with suspected infection upon ICU admission (*p* < 0.004 for all). Figure 2 presents the AUROCs for each of the scores and the model among this sub-group. Again, MEWS performed the best in our cohort with an AUROC of 0.73 (95% CI 0.65–0.81), followed by R-MPM at 0.72 (95% CI 0.64–0.80). The others were qSOFA, 0.63 (95% CI 0.55–0.71); UVA 0.69 (95% CI 0.64–0.75).

**TABLE 3:** Distribution of mortality scores and association with hospital mortality among patients with suspected infection.

Predictive score or model	All *N* = 163	Survived *N* = 95	Died *N* = 68	*p*-value
**MEWS**				
Median (IQR)	5 (3–7)	4 (3–6)	6 (5–8)	0.001
≥5 points, *n* (%)	91 (56)	38 (40)	53 (78)	<0.001
**UVA**				
Median (IQR)	5 (4–6)	4 (2–6)	5 (4–6)	<0.001
Low risk, *n* (%)	25 (15)	23 (24)	2 (3)	<0.001
Medium risk, *n* (%)	56 (34)	36 (38)	20 (29)	0.2
High risk, *n* (%)	82 (50)	36 (38)	46 (68)	<0.001
**qSOFA**				
Median (IQR)	1 (1–2)	1 (1–2)	2 (1–2)	0.005
≥1 point, *n* (%)	148 (91)	81 (85)	67 (99)	0.004

Percentages were calculated with the column total as the denominator. Suspected infection defined as those receiving IV antibiotics within 24 h of ICU admission. R-MPM does not have a score as it is a model that requires regression calculation. IQR, Interquartile range; MEWS, Modified early warning score; UVA, Universal vitals assessment; qSOFA, quick sequential organ failure assessment.

## Discussion

In this study, we assessed the predictive capacity for hospital mortality of four severity of illness scores and a regression model in our resource-limited ICU setting. Due to the large percentage of missingness for TropICS we decided not to use it in the final comparison. Using previously published thresholds for the remaining three scores, we found that patients with scores above these values had a significantly higher probability of death than those with scores below. When using AUROC, we found all four scores and the model to have reasonable discrimination, with MEWS performing best with AUROC of 0.76.

The scores and model also varied in availability of the component variables; R-MPM had the lowest rate of patients with missing at least one variable (*n* 15, 4%), while TropICS had the highest rate (*n* 284, 84%). This is an important consideration in choosing which score or model to use in a resource-constrained setting where laboratory values are often not readily available.

Our findings are consistent with the original studies detailing the development and validation of the scores. Like Kruisselbrink et al. (8) we found that a score of ≥5 was associated with an increased risk of death. Despite MEWS being studied in ward patients in an Ugandan national referral hospital, the similarities of ICU bed limitation make this score an appropriate tool for our setting. A UVA score of ≥4 was associated with increased mortality in the initial development study (9). We also noted an increased risk of mortality in those with scores ≥4, but UVA did not perform as well when comparing AUROC. A prospective cohort study conducted in two hospitals in Tanzania showed that UVA performed favorably compared to MEWS, SIRS, and qSOFA in predicting in-hospital mortality among patients with a febrile illness (19). One reason that our findings may have differed was the fact that only 3% of the patients in that study were missing data, as clinical assessment and laboratory evaluation as performed prospectively by the study team.

The utility of qSOFA has been questioned as a screening tool and as a means of risk stratification due to its low sensitivity. Separate studies have suggested using a score of ≥1 in order to improve upon this (10, 23). Making this adjustment to our cohort did show that it could be used to predict mortality in those presenting with a suspected infection. However, when compared to the other tools, the discrimination was not as good.

R-MPM had a reasonable AUROC in our study, though not as high as in its derivation population (11). The variables for R-MPM were largely available. However, the need for repeat regression calculations may make this difficult to use in real time at the bedside.

An important strength of this study is that it is the first to directly compare all scores previously used in resource limited settings. In addition, it describes the availability and missingness of the variables needed for each model (24). Another strength is the rural study setting, which is where the majority of sub-Saharan Africans reside (25). Our ICU in rural Kenya is representative of ICUs in low resource settings. Lastly, consecutive enrollment of a mixed medical and surgical population broadens the generalizability of our findings.

This study has several limitations. First, it was limited to a single center. Second, we found data for variables to be missing, even with a prospective study design. While this is a limitation, it also reflects the reality of what variables are likely to be available in similar resource limited settings. The frequency of missing data itself is an important result that can help drive decisions about which tool to use in a given setting (24). Third, due to the limited number of ICU beds available in our hospital as compared with demand, many critically ill patients would not have been admitted to the ICU. This could lead to a selection bias of unclear direction.

## Conclusion

In this single-center, prospective observational cohort study, we compared the ability of four scores and a regression model to predict mortality in a resource limited adult ICU in Kenya. Each proved capable of predicting mortality in our patient population. There was wide variability in the availability of component variables for each score. Any of the scores might be a reasonable choice for use in a resource limited setting, with consideration to which variables are readily available as part of clinical care in a given setting.

## Data availability statement

The data supporting the conclusions of this article are available upon request to the authors, without undue reservation.

## Ethics statement

Approval for this study was granted by the AIC Kijabe Hospital IRB committee and was granted a waiver of exemption.

## Author contributions

BB, ER, and KR participated in the development of the study, creation of the protocol, and supervision of the project. BB, MJ, and KR wrote the manuscript. BB, MJ, GO, HG, EK, SW, TN, and SL participated in the collection and validation of the data. SL was responsible for the creation of the local application (Banda Health). BB and KR led and GO, EK, SW, TN, and MJ participated in the data analysis. All authors critically reviewed the manuscript and approved the final submitted version.

## Footnotes

1
https://www.bandahealth.org
