# Supplementary material for: Association of clinical prediction scores with hospital mortality in an adult medical and surgical intensive care unit in Kenya
Source: Front Med (Lausanne). 2023 Apr 5;10:1127672. doi: 10.3389/fmed.2023.1127672 (PMC10113620; doi:10.3389/fmed.2023.1127672)
Supplement: Supplementary file 1 [file Data_Sheet_1.docx]

**e-Supplement Table 1. Variables Collected and Percent Missing**

| Variable | No. Missing (%) |
| --- | --- |
| Total patients included | 338 |
| Age | 0 (0) |
| Gender | 0 (0) |
| Systolic Blood Pressure | 75 (22) |
| Heart Rate | 14 (4) |
| Respiratory Rate | 15 (4) |
| Temperature | 76 (23) |
| GCS/AVPU | 14 (4) |
| Oxygen Saturation | 82 (24) |
| Suspected infection | 14 (4) |
| Emergency surgery | 0 (0) |
| BUN | 253 (75) |
| Hemoglobin | 90 (27) |
| HIV status | 106 (31) |
| MEWS | 77 (23) |
| UVA | 85 (25) |
| TropICS | 284 (84) |
| qSOFA | 76 (23) |
| RMPM | 15 (4) |

Variable from Tables 1 and 2 and the percentage of missing data for each variable. The percentages were calculated out of the total denominator of 338. A score is listed as missing if the patient was missing any one component of the score. Abbreviations: GCS, Glasgow Coma Scale; AVPU, Alert Verbal Pain Unresponsive; BUN, blood urea nitrogen; HIV, Human Immunodeficiency Virus; MEWS, Modified Early Warning Score; UVA, Universal Vitals Assessment; TropICS, Tropical Intensive Care Score; qSOFA, quick Sequential Organ Failure Assessment; R-MPM, Rwanda Mortality Prediction Model.

**e-Supplement Table 2. Composition of Scores and Model**

|  | **R-MPM** | **MEWS^ƚ^** | **TropICS** | **UVA^ǂ^** | **qSOFA** |
| --- | --- | --- | --- | --- | --- |
| Variable | Age | SBP | RR | Temp | RR> 22 |
|  | Infection | HR | SBP | HR | SBP <100 |
|  | Low BP/shock (SBP < 90 mmHg) | RR | Emergency surgery as reason for ICU admission | RR | GCS ≤ 14 |
|  | GCS | Temp | GCS | BP |  |
|  | HR | AVPU* | BUN | SpO2 |  |
|  |  |  | Hb | GCS |  |
|  |  |  |  | HIV status |  |
| Method of calculation | OR for each decile | Summation of variables; range  0-14 | Summation of scores assigned for each variable;  % risk calculated using nomogram | Summation of scores; range 0-13; stratified to low, medium, high risk based upon total score | Summation of variables; range 0-3 |
| Definition of a ‘positive’ score | Regression calculated for each patient to determine AUROC | Score ≥5 defined as critically ill | Sum, extrapolated to % mortality | Score >4 defined as high risk | ≥1 |

Abbreviations: R-MPM, Rwanda Mortality Prediction Model; MEWS, Modified Early Warning Score; TropICS, Tropical Intensive Care Score; UVA, Universal Vitals Assessment; qSOFA, quick Sequential Organ Failure Assessment; SBP, Systolic Blood Pressure; RR, Respiratory Rate; Temp, Temperature; HR, Heart Rate; BP, Blood Pressure; GCS, Glasgow Coma Scale; AVPU, Alert Verbal Pain Unresponsive; BUN, Blood Urea Nitrogen; Hb, Hemoglobin; SpO2, peripheral oxygen saturation; OR, odds ratio;

^ƚ^MEWS involves vital signs parameters that are assigned a numerical score depending on the severity of aberration from the norm. ^ǂ^UVA is calculated by adding the assigned numerical scores based on severity of aberration from the norm.

**e-Supplemental Figure 1. TropICS Nomogram**

**
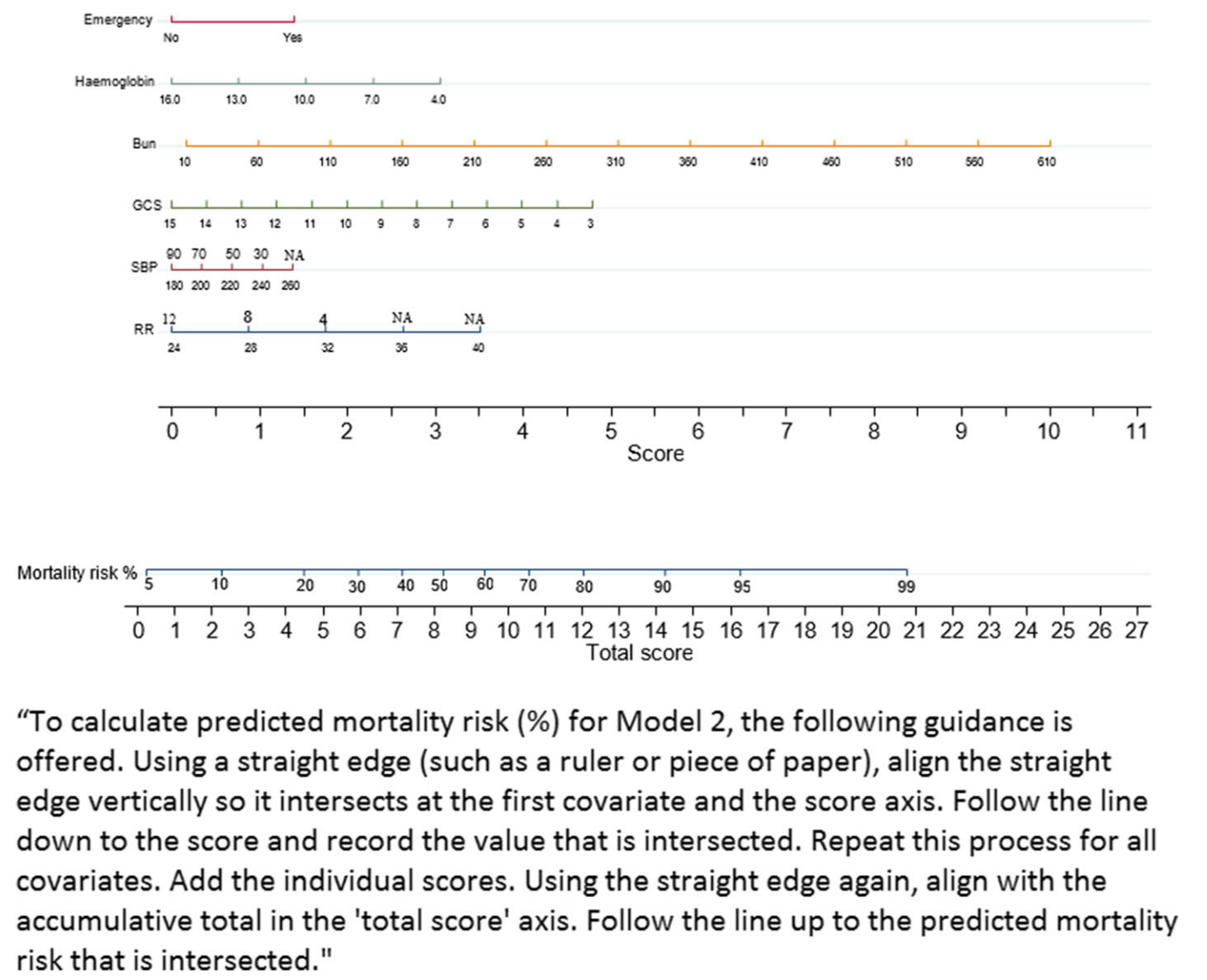
**

Adapted from Haniffa et al. (11)
